# Kidney and pregnancy outcomes in pregnancy-associated atypical hemolytic uremic syndrome: A systematic review and meta-analysis

**DOI:** 10.1097/MD.0000000000041403

**Published:** 2025-01-31

**Authors:** Priti Meena, Ruju Gala, Rashmi Ranjan Das, Vinant Bhargava, Yellampalli Saivani, Sandip Panda, Alok Mantri, Krishna Kumar Agrawaal

**Affiliations:** a Department of Nephrology, All India Institute of Medical Sciences, Bhubaneswar, Odisha, India; b Department of Nephrology, Zynova Shalby Hospital, Mumbai, Maharashtra, India; c Department of Pediatrics, All India Institute of Medical Sciences, Bhubaneswar, Odisha, India; d Department of Nephrology, Sir Ganga Ram Hospital, New Delhi, India; e Department of Nephrology, Santhiram Medical College, Nadyal, Andhra Pradesh, India; f Department of Gastroenterology, Kalinga Hospital, Bhubaneswar, Odisha, India; g Department of Nephrology, Universal College of Medical Sciences, Bhairahawa, Siddhartha Nagar, Nepal.

**Keywords:** acute kidney injury, atypical hemolytic uremic syndrome, dialysis, eculizumab, end-stage kidney disease, pregnancy

## Abstract

**Background:**

Pregnancy-associated atypical hemolytic uremic syndrome (p-aHUS) is a rare, life-threatening condition characterized by microangiopathic hemolytic anemia, thrombocytopenia, elevated liver enzymes, and acute kidney injury. Prompt diagnosis and therapy are crucial due to the high risk of progression to chronic kidney disease (CKD), end-stage kidney disease (ESKD), and dialysis dependency, as well as significant maternal and fetal morbidity and mortality.

**Methods:**

A comprehensive literature search was conducted across EMBASE, MEDLINE, and the Cochrane CENTRAL from January 2000 to March 2024. Studies reporting on pregnancy and kidney outcomes in women diagnosed with p-aHUS were included.

**Results:**

Ten studies involving 386 pregnancies in 380 patients met the inclusion criteria for the final analysis. Renal outcomes varied, with mean creatinine levels ranging from 0.72 to 8.734 mg/dL. Dialysis was required in 66.6% of patients, and 25% developed ESKD. Maternal complications included preeclampsia (36.4%) and hemolysis, elevated liver enzymes, and low platelets syndrome (29.7%), with a 5% maternal mortality rate. Fetal complications included intrauterine fetal demise (n = 25), intrauterine growth restriction, low birth weight, and prematurity. Treatment with eculizumab significantly reduced the risk of CKD and ESKD, with a pooled risk ratio of 0.20 (95% confidence interval: 0.09–0.44) and low heterogeneity (*I*² = 0%, *P* = .43).

**Conclusion:**

This analysis highlights the severe kidney and pregnancy outcomes associated with p-aHUS. Eculizumab treatment is significantly beneficial in reducing the risk of CKD and ESKD.

## Graphical abstract

**Figure:**
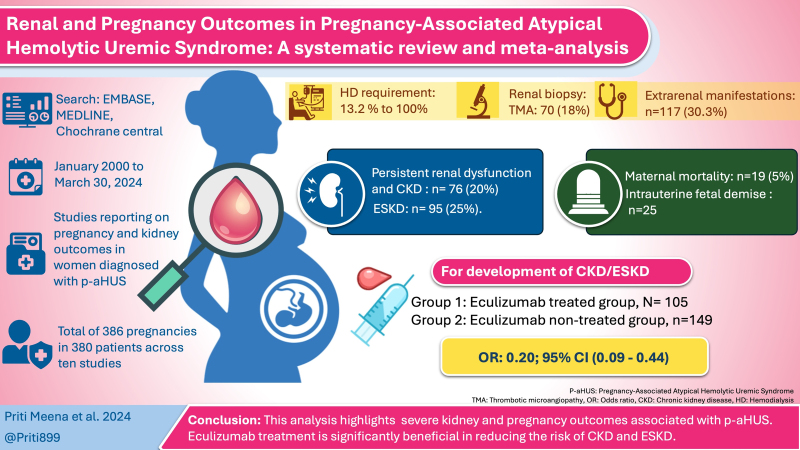

## 1. Introduction

Pregnancy-associated atypical hemolytic uremic syndrome (p-aHUS) is a rare condition characterized by microangiopathic hemolytic anemia, thrombocytopenia, elevated liver enzymes, and acute kidney injury.^[1]^ The occurrence rate of atypical hemolytic uremic syndrome (aHUS) is approximately estimated at 0.23 cases per year per million individuals, with variations observed across different populations.^[2,3]^ About 10% to 20% of diagnosed cases of aHUS arise during pregnancy.^[2]^ Apart from sepsis and hypertensive complications, p-aHUS is one of the significant causes of acute kidney injury during pregnancy. Triggered by pregnancy, genetically predisposed women can develop HUS, characterized by diffuse endothelial damage and platelet consumption.^[1]^ In a retrospective study by Bayer et al on a French cohort with identified thrombotic microangiopathies (TMAs) cases, it was found that pregnancy constituted the primary cause of secondary TMA, accounting for 35% of cases.^[4]^ p-aHUS is a life-threatening condition that requires prompt diagnosis and therapy. The management of p-aHUS is a major clinical challenge. Pregnancy-associated aHUS often progresses to chronic kidney disease (CKD), end-stage kidney disease (ESKD), dialysis, or kidney transplant.^[5,6]^ p-aHUS can also result in high maternal and fetal morbidity and mortality. Nonetheless, information regarding the outcomes of p-aHUS is scarce, primarily derived from case reports and case series.^[5]^ Plasma exchange is predominantly used in treating p-HUS with varying results. Eculizumab, a monoclonal antibody targeting complement protein C5, has proven effective in treating aHUS.^[7]^ It is now also increasingly used in cases of p-aHUS, yet data remains limited. A meta-analysis of the available literature on this topic can help to provide a more comprehensive understanding of the association between p-aHUS, pregnancy, and kidney outcomes.

## 2. Methodology

### 2.1. Literature search

A comprehensive literature search was conducted across EMBASE, PUBMED/MEDLINE, and the Cochrane CENTRAL from January 2000 to March 30, 2024, without language or geographic restrictions. The aim was to assess renal and pregnancy outcomes in women with p-aHUS. The literature search was done using a predefined search strategy incorporating terms such as “kidney,” “renal,” “pregnancy,” “obstetrics,” and “atypical hemolytic uremic syndrome. Detailed search strategy for the analysis is provided in Supplemental Digital Content, File 1, http://links.lww.com/MD/O319. Additionally, manual screening of references in included articles was conducted to identify potentially relevant studies. The study adhered to the PRISMA statement guidelines. The systematic review protocol has been registered in PROSPERO (registration number: CRD42024500717; http://www.crd.york.ac.uk/PROSPERO).

This is a meta-analysis and did not require ethical approval.

### 2.2. Selection criteria

The eligible studies included: (1) Clinical trials or observational studies (cohort, case-series, or cross-sectional) reporting on pregnancy and kidney outcomes in women diagnosed with p-aHUS, and (2) The studies must be published as full-text articles. They must provide the data on kidney outcomes.

Exclusion criteria were: (1) Studies that report on nonhuman subjects or animal models, (2) Studies that report on cases of p-aHUS caused by a secondary cause, such as medication or infection, and (3) Conference abstracts, review articles or editorials.

### 2.3. Study selection

**Figure 1.**
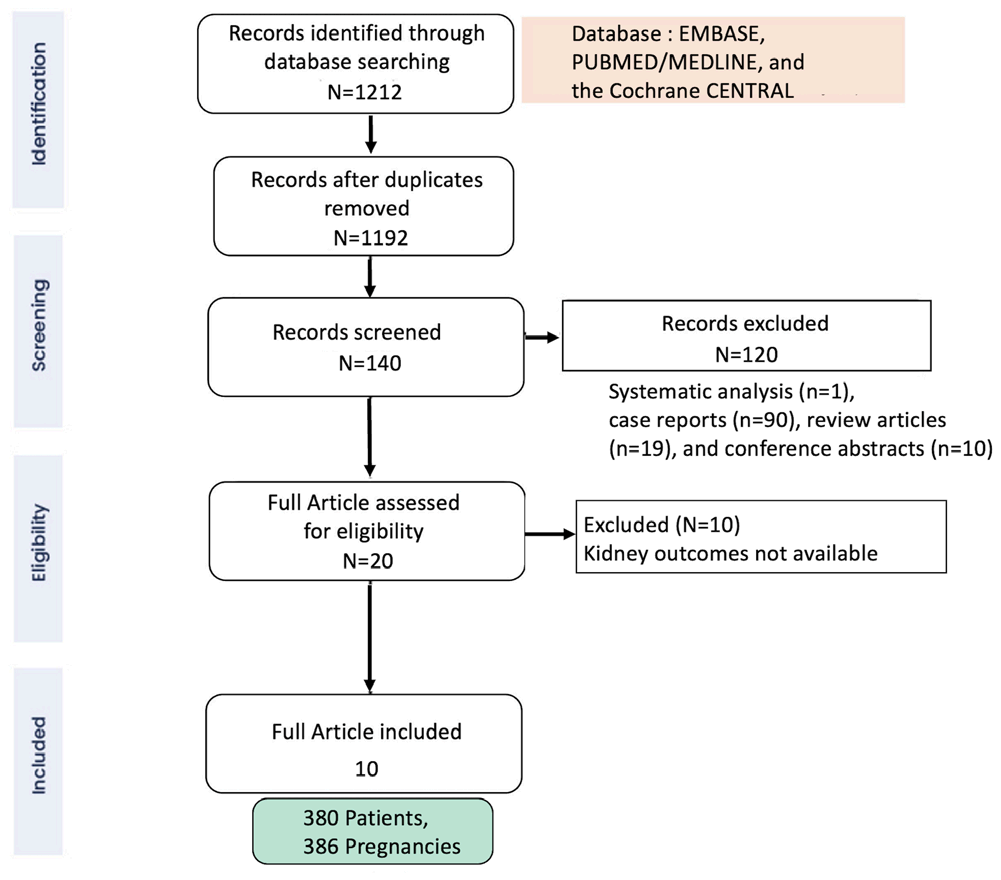
The PRISMA flowchart. PRISMA = preferred reporting items for systematic reviews and meta-analysis.

The detailed process has been provided as a PRISMA flow chart (Fig. 1). After the removal of duplicate records, titles and abstracts were independently reviewed by 2 reviewers (PM and VB) to assess study eligibility. Full-text screening followed, resolving any discrepancies through consensus with the third author (SP). In cases of multiple publications from the same study, the most comprehensive or latest updated dataset was selected. Articles deemed unsuitable based on title and/or abstract consensus were excluded. Full-text articles underwent independent screening by 2 authors (RG and SY), with inclusion requiring mutual agreement. In instances of initial disagreement, a third author was consulted for resolution through discussion. Data extraction was performed by 2 authors using a predesigned form, with verification by 2 additional independent authors (SP and RD).

### 2.4. Data extraction

A structured data collection form was utilized to systematically gather pertinent information from each included study. The collected data comprised various information including name of the first author, publication year, demographic characteristics of patients such as age, gestational period of pregnancy at the time of p-aHUS diagnosis, parity, recurrence, family history, kidney functions at the time of diagnosis, need for dialysis, availability of kidney biopsy data, kidney outcomes (including development of CKD/ESKD and dialysis dependence), maternal outcomesh (such as presence of hemolysis, elevated liver enzymes, and low platelet count syndrome [HELLP], hypertension, preeclampsia, bleeding, extrarenal complications, and mortality), and fetal outcomes (including growth retardation, intrauterine deaths, neonatal deaths, prematurity, and low birth weights) and details of treatments received such as eculizumab and plasma exchange, along with their respective outcomes.

### 2.5. Risk of bias and certainty of evidence assessment

The Newcastle–Ottawa Quality Assessment Scale was used to evaluate the quality of observational studies, while the Cochrane Risk-of-Bias Tool was utilized for clinical trials. A comprehensive assessment of each study is provided in Supplemental Digital Content, File 2 in New Ottawa scale (Table S1, http://links.lww.com/MD/O319), detailing the strengths and limitations identified through quality appraisal methods. This assessment was conducted independently by 2 reviewers (PM and SP), with any discrepancies resolved through discussion. The certainty of evidence for each outcome was determined using the “Grading of Recommendations Assessment, Development, and Evaluation” methodology.

### 2.6. Data synthesis and statistical analysis

The ReviewManager 5.4 (RevMan) Meta-Analysis software, was used for all statistical analyses. For continuous outcomes, the results were expressed as standardized mean difference with 95% confidence interval (CI). Dichotomous outcomes were presented as odds ratios (ORs) with their associated 95% CI. A *P*-value of <.05 was taken as statistically significant. Given the potential for between-study variability, a random-effects model was employed instead of a fixed-effect model. Between-study heterogeneity was assessed using Cochrane *Q* test and the *I*^2^ statistic. An *I*^2^ value of 0% to 25% indicates insignificant heterogeneity, 26% to 50% denotes low heterogeneity, 51% to 75% signifies moderate heterogeneity, and 76% to 100% suggests high heterogeneity. Publication bias was evaluated through both funnel plots and Egger regression test.

### 2.7. Study selection

The initial search yielded 1212 articles, after removing duplicates (n = 20), 1192 studies underwent title and abstract screening. The full texts of 140 articles were retrieved for eligibility assessment, and 120 articles were excluded for the following reasons: systematic analysis (n = 1), case reports (n = 90), review articles (n = 19), and conference abstracts (n = 10). Finally, a total of 386 pregnancies in 380 patients across 10 studies were included in the final analysis.^[8–17]^

### 2.8. Characteristics of the study population

The overall prevalence of p-aHUS varied among studies ranging from 9% to 26%.^[9,11]^ In the Global aHUS Registry, out of 1858 patients, 51 (2.7%) were identified with p-aHUS.^[15]^ Baseline characteristics of the included studies are summarized in Table 1. Five studies^[10,11,13,14,17]^ were retrospective 2 studies^[8,12]^ were prospective and one^[15]^ was the analysis of data from the global aHUS registry. The mean age of the patients ranged from 26.28 to 32 years across studies. The majority of patients were primigravida with frequency varying from 36% to 90%. Fifty-three (13.9%) had a recurrence of aHUS, reported in 6 studies.^[7–10,15,16]^ Family history of aHUS was present in 25 (6.5%) cases.^[10,15,16]^ Eleven patients were kidney transplant recipients,^[9,16]^ and one^[11]^ had undergone combined pancreas–kidney transplantation.

**Table 1:** Characteristics of the study population.

Sl No.	Author	Year	Study design	Total number of patients (n)	Mean age in years ± SD	Primi, n (%)	Ante-partum, n (%)	POG in weeks	Postpartum, n (%)	Recurrence, n (%)	Family history of aHUS, n (%)	KTP, n (%)
1.	Servais et al	2016	Prospective	6 pregnancies in 3 patients of aHUS	28.5	NA	4 (66.6)	29.4 ± 4.9	-	4 (66.6)	NA	NA
2.	Gaggl et al	2016	Prospective and retrospective	14 women with aHUS who had pregnancies were included. 7 (26%) were complicated p-aHUS	29 ± 12	5 (35.7)	4 (28.5)	NA	2 (14.2)	7 (50)	NA	4 (28.5%)
3.	Bruel et al	2017	Retrospective	87	29 ± 6	48 (58)	20 (24)	–	63 (76)	36 (42)	14 (16%)	None
4.	Huerta et al	2018	Retrospective	22	32 ± 6.5	16 (73)	6 (27)	25.2 (4–36) wk	16 (73)	4 (18)	None	1 combined PKT
5.	Ramchandran et al	2018	Prospective	21	26.38 ± 4.33	NA	None	NA	21 (100)	NA	NA	NA
6.	Naqvi et al	2020	Retrospective	49	29.02 ± 5.258	NA	2 (4)	NA	47 (95.9)	NA	NA	NA
7.	Timmermans et al	2020	Retrospective	7	NA	NA	3 (42)	NA	4 (57.1)	NA	NA	NA
8	Fakhouri et al	2021	Data from global aHUS registry	51	31.2 ± 5.9	50 (90)	28 (54.9)	0–12 wk: 12 (23.5%), 13–24 wk, 2 (3.9%) >24 wk: 14 (27.5)	23 (45.1)	1 (2.0)	6 (11.8)	None
9.	Rondeau et al	2022	Prospective and retrospective	44 pregnancies in 41 patients	Median age reported: 27.6	26 (63.4)	TMA developed in 3 (6.8%) in antipartum period	NA	1 (2.27)	1 (2.27)	5 (16.1)	7 (17)
10	Korotchaeva et al	2024	Retrospective	85	28.5 (24.5–33.0) in eculizumab group and 29.0 (25.0–36.0) in non-eculizumab group	NA	None	NA	85 (100)	None	NA	NA

aHUS = atypical hemolytic uremic syndrome, KTP = kidney transplant recipient, NA = not sure/not reported, PKT = combined pancreatic kidney transplantation, POG = period of gestation, SD = standard deviation.

### 2.9. Renal outcomes

Renal outcomes varied among studies, with mean creatinine levels ranging from 0.72 to 8.734 mg/dL (see Table 2). Dialysis was required in a substantial proportion of patients, with rates ranging from 13.2 % to 100%. Overall, out of 342 (excluding a study by Rondeau et al^[16]^ that included 3 (6.8%) chronic hemodialysis patients) 228 patients (66.6%) required dialysis. Renal biopsies were done in 72 (19%), TMA was seen frequently in 70 (18%), and in 2 cases C3 glomerulonephritis was reported. Most studies had a follow-up of at least 6 months. Outcomes after a follow-up duration of 16 years were reported in 1 study.^[14]^ Seventy-six patients (20%) were found to have persistent renal dysfunction and developed CKD. ESKD was reported in 95 patients (25%).

**Table 2:** Kidney presentation and outcomes.

Sl no.	Author	Mean/median creatinine in mg/dL ± SD	Kidney biopsy	Dialysis requirement at presentation, n (%)	Follow-up	Kidney outcome, n (%)	
						CKD development	Dialysis dependency/ESKD
1.	Servais et al	1.49 ± 0.5	n = 1: TMA	1 (16.6)	Mean 157.4 mo	6 (100%) had eGFR 25–45	None
2.	Gaggl et al	NA	n = 1: TMA	None	Median 8.5 years (3–21)	4 (28.5%) CKD	3 (21.4), 1 (7.1%) received a renal allograft
3.	Bruel et al	6.1 ± 5.2	n = 8 (9.1%) All showed TMA	56 (71)	7.2 years	15 (19%) progressed to CKD.	41 (53)
4.	Huerta et al	3.5 (IQR: 2.3, 4.23)	n = 11 (50%) showed TMA in all except 1. An associated C3GN was seen in n = 2 and n = 1 showed immune complex MPGN	9 (41)	Average 7.9 y/patient	6 (27.2) progressed to CKD.	6 (27.2)
5.	Ramchandran et al	4.3 ± 2.2	n = 1: TMA	21 (100)	At least 6 mo	2 (9.5%) had persistent kidney dysfunction	14 (66.7)
6.	Naqvi et al	8.734 ± 4.027	n = 49 (100%), had TMA, n = 21 (43.7%): showed TMA with focal CN, n = 1 (20.8%) had diffuse CN, n = 1 with ATN	49 (100)	More than 90 d	16 (32.7%) Persistent kidney dysfunction	17 (34.7)
7.	Timmermans et al	Median 5.52 (2.9–6.9)	n = 1: TMA	NA	16 years	3 (42%) persistent kidney dysfunction	1 (14.2)
8.	Fakhouri et al	NA	NA	7 (13.7)	3.73 ± 2.01 y	NA	2 (3.9%) progressed to ESKD
9.	Rondeau et al	0.72 (0.48–2.26)	NA	Study included 3 (6.8%) chronic HD patients	NA	3 (6.8%) chronic HD patients were continued on HD No worsening of eGFR reported in KTP	None (excluding chronic HD pts)
11.	Korotchaeva et al	5.64 (4.14–7.24) in eculizumab group and 4.44 (2.94–5.54)	NA	85 (100)	NA	24 (33.3%) in surviving 72 patients	11 (14)

ATN = acute cortical necrosis, C3GN = C3 glomerulonephritis, CKD = chronic kidney diseases, CN = cortical necrosis, ESKD = end-stage kidney diseases, eGFR = estimated glomerular filtration rate, HD = hemodialysis, KTP = kidney transplant recipient, MPGN = membranoproliferative glomerulonephritis, NA = not available, SD = standard deviation, TMA = thrombotic microangiopathy.

### 2.10. Maternal and fetal complications

Maternal complications during pregnancy included preeclampsia in 62 out of 170 cases (36.4%) and HELLP syndrome in 36 out of 121 cases (29.7%). Maternal mortality during episodes of aHUS was low across studies, with maternal deaths reported in 19 cases (5%) (see Table 3). Extrarenal manifestations were reported in 117 patients (30.3%). Severe hemorrhage was reported in 14 patients (1.6%). Additionally, a study by Korotchaeva et al reported extrarenal manifestations in a significant proportion of patients (95%) including acute respiratory distress syndrome (54%), coma (20%), acute heart failure (12%), stroke (6%), and myocardial infarction (1%).^[17]^ Intrauterine fetal demise was reported in 25 cases. Other fetal complications were intrauterine growth restriction, low birth weight, and prematurity.

**Table 3:** Maternal and fetal complications.

Sl no.	Author	Abortion, n (%)	Pre-eclampsia, n (%)	HELLP, n (%)	HTN, n (%)	Maternal mortality, n (%)	Maternal complication, n (%)	Fetal complications, n (%)
1.	Servais et al	1 (16.6)	2 (33.3%)	1 (16.6)	3 (50)	None	1 (16.6): Bleeding	1 (16.6%): IUD, 2 (33.3%): growth retardation
2.	Gaggl et al	4 (28.5)	2 (14.2)	1 (7.1)	2 (14.2)	None	NA	2 (14.2) preterm live births and 2 (14.2) stillborn
3.	Bruel et al	NA	NA	NA	NA	None	7 (9) had extrarenal manifestation	11 (14%) fetal death
4.	Huerta et al	1	17 (77.2)	7 (31.8)	19 (86)	None	n = 3, (13.6): Severe bleeding	2 (9.5) LBW
5.	Ramchandran et al	None	7 (33.3)	9 (42.8)	NA	5 (23.8)	9 (42.8) had postpartum hemorrhage	NA
6.	Naqvi et al	None	NA	NA	NA	1 (2)	1: Hemiparesis	11 (22) IUD
7.	Timmermans et al	NA	4 (57.1)	1 (14.2)	NA	None	1 (14.2): Bleeding	1 (12.5) neonatal death and 3 (37.5) SGA
8.	Fakhori et al	NA	28 (54.9)	17 (33.3)	NA	None	30 (58.8%) had extra renal manifestations. Cardiovascular: n = 7 (13.7), pulmonary: n = 5 (9.8) CNS: n = 9 (17.6), and gastrointestinal: n = 9 (17.6)	NA
9.	Rondeau et al	4 (9.1)	2 (4.5)	NA	5 (11.3)	None	NA	Premature births: 8 (34.8), late fetal death: 1 (2.3), and LBW: 9 (20.4)
10.	Korotchaeva et al	NA	NA	NA	NA	13 (12.9)	ARDS: 46 (54%), coma: 17 (20%), acute heart failure: 10 (12%), stroke:5 (6%) and myocardial infarction: 1 (1%)	NA

ARDS = acute respiratory distress syndrome, aHUS = atypical hemolytic uremic syndrome, CNS = central nervous system, IUD = intrauterine deaths, HELLP = hemolysis, elevated liver enzymes, and low platelets, HTN = hypertension , LBW = low birthweight, NA = not available/not sure, SGA = small for gestational age.

### 2.11. Treatment outcome

**Figure 2.**
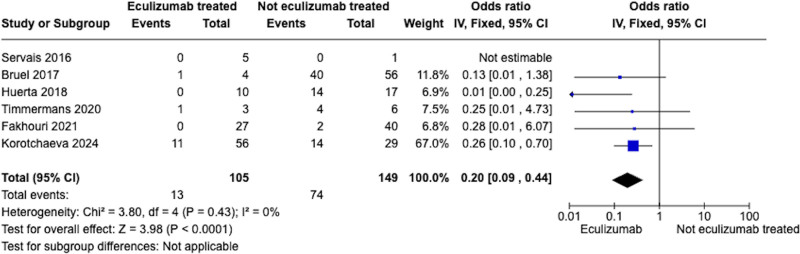
Forrest plot evaluating the estimated proportions (95% CI) for the development of chronic kidney diseases in eculizumab-treated and nontreated patients. Figure: Visual abstract of the study. CI = confidence interval.

In the meta-analysis, 105 (n = 27.2%) of patients received eculizumab (see Table 4). The timing for receiving the first eculizumab dose after the onset of aHUS varied among studies, ranging from 4 days to 2 months. Figure 2 shows the estimated proportions (95% CI) for the development of chronic kidney disease in eculizumab-treated and nontreated patients. A total of 6 studies were analyzed in meta-analysis with a total of 105 subjects in the eculizumab-treated group and 149 subjects in the eculizumab nontreated group. Based on the analysis performed using the random-effects model with Mantel–Haenszel method to compare the OR, a statistically significant difference was noted between the 2 groups [OR 0.20; 95% CI 0.09–0.44]. The heterogeneity among the 6 included studies was low (*I*^2^ = 0%, *P* = .43). In the study by Rondeau et al,^[16]^ 24 patients (54.5%) were already exposed to eculizumab, while in the study by Sevais et al,^[8]^ 5 patients (83.3%) were on eculizumab maintenance therapy. In the cohort by Fakhouri et al, the response rate was 100%.^[15]^ The risk of ESKD was significantly higher for women not treated with eculizumab compared to those treated with eculizumab. The unadjusted hazard ratio was 0.14 (95% CI 0.04–0.47; *P* = .002). Similarly, in the cohort by Korotchaeva et al,^[17]^ induction therapy with eculizumab resulted in a 74% reduction in the risk of the composite primary endpoint, which included death and ESKD, and an 89% reduction in the risk of death from all causes compared to plasma therapy alone. None of the studies reported differences in maternal and fetal complications; therefore, the meta-analysis was performed solely for renal outcomes.

**Table 4:** Treatment outcomes of the study populations.

Sl no	Author	Eculizumab, n (%)	Median time of receiving first eculizumab dose from onset of aHUS	Response to eculizumab, n (%)	Plasmaphereses, n (%)	Response to plasmaphereses, n (%)
1.	Servais et al	5 (83.3)	On maintenance	Partial recovery in all	1 (16.6)	1 (100)
2.	Gaggl et al	Not given	Not given	Not given	2 (14.2)	1 (50)
3.	Bruel et al	4 (5)	Given in 4 patients at 4 d, 5 d, 1 mo, and 2 mo after aHUS diagnosis	3 (75) had complete recovery	56 (78)	16 (28.5)
4.	Huerta et al	10 (45)	17 d (IQR: 8, 23)	10 (100)	17 (77)	3 (17.6)
5.	Ramchandran et al	Not given	Not given	Not given	Not given	Not given
6.	Naqvi et al	Not given	Not given	Not given	37 (75.5%)	NA
7.	Timmermans et al	3 (42)	NA	2 responded	6 (85.7)	2 (33.3)
8.	Fakhouri et al	27 (52.9)	0.07 ± 0.13 years	Response in 100% The risk of ESKD was significantly higher for women not treated with eculizumab, compared with eculizumab-treated women The unadjusted HR was 0.14 (95% [CI] 0.04, 0.47; *P* = .002) Mean eGFR improvement after eculizumab treatment, with a increase relative to baseline: 56.2 ± 39.8	40 (78.4)	38 (95) HR adjusted for dialysis, PE/PI treatments, and at the time of initial TMA: 0.08 (95% CI 0.01, 0.65; *P* = .019)
9.	Rondeau et al	24 (54.5) were eculizumab exposed, 3 (6.8%) not exposed pt received as treatment of TMA	NA	NA	NA	NA
10.	Korotchaeva et al	56 (65.8)	Early (initiated within 7 d after disease onset) in n = 28 (33), delayed (on day 8 to 20) in n = 15 (17.6) patients and late (on day 21 or more) in n = 13 (15.2) patients	Induction therapy with eculizumab resulted in a 74% reduction in the risk of composite primary endpoint that included death and ESKD and a 89% reduction in the risk of death from all causes as compared to plasma therapy alone	death or chronic kidney renal failure requiring regular dialysis treatment) in 14 (48.2%) of 29 patients treated without eculizumab	NA

aHUS = atypical hemolytic uremic syndrome, CI = confidence interval, eGFR = estimated glomerular filtration rate, ESKD = end-stage kidney diseases, HR = hazard ratio, NA = not available/not sure, PE = Plasmaphereses, PI = plasma infusion, TMA = thrombotic microangiopathy.

Figure 2 shows a forrest plot evaluating the estimated proportions (95% CI) for the development of chronic kidney diseases in eculizumab-treated and nontreated patients.

### 2.12. Safety outcome with eculizumab

All studies reported that the treatment of p-HUS with eculizumab was well tolerated. No patients had allergic reactions or infections. No studies indicated that death was related to the use of eculizumab. No congenital abnormalities in fetuses were reported.

### 2.13. Evaluation for publication bias

We found no significant publication bias as assessed by the funnel plots and Egger regression asymmetry test for the rates of chronic kidney disease development in eculizumab treated and nontreated patients. The funnel plot should ideally be constructed if we have at least 10 studies. Here the funnel plot looks asymmetrical suggesting a chance of publication bias, but we cannot confirm it as studies are fewer in number. See Supplemental Digital Content, Figure S1, http://links.lww.com/MD/O319, showing funnel plot diagram of the involved studies.

### 2.14. Quality assessment of observational studies

The Newcastle–Ottawa scale was used. The Newcastle–Ottawa scale scores of each study ranged from 5 to 7, with an average of 6.1, which means the studies were of average to good quality. Detailed information is in Supplemental Digital Content, File 2, New Ottawa scale (Table S1, http://links.lww.com/MD/O319).

## 3. Discussion

Our study has analyzed data from 380 patients who developed atypical HUS during their pregnancy or in the postpartum period (p-aHUS). To the best of our knowledge, this is the most updated meta-analysis to consolidate the long-term pregnancy and renal outcomes of women with p-HUS. These patients of pregnancy aHUS account for 2.7% of all the patients affected by aHUS globally, amounting to 1858 patients as notified by the global aHUS registry.^[15]^ This meta-analysis has included data on all possible pregnancy aHUS (primipara as well as multipara women), including kidney transplant recipients and a kidney pancreas transplant recipient.

In this current meta-analysis of 10 published studies of 380 patients with 386 events of p-aHUS, we found that aHUS complicating pregnancies has disastrous maternal and fetal outcomes, which can be ameliorated with the use of complement inhibitors such as eculizumab. Eculizumab showed better treatment outcomes as compared to conventional modalities such as plasmapheresis, plasma infusion, and immunosuppression like corticosteroids. The patients treated with eculizumab had a 74% lesser risk of progression to ESKD and dialysis-dependent kidney failure. A significant mortality benefit was also observed in patients receiving eculizumab.

Clinical trials and case reports have shown eculizumab effectively treats complement-mediated aHUS in nonpregnant individuals, improving hematological and renal parameters.^[18,19]^ The successful treatment outcomes of pregnancy aHUS with the administration of eculizumab as shown in our meta-analysis reinforce our understanding of p-aHUS as a complement-mediated TMA.

A significant number of pregnancies with aHUS had a background of associated obstetric complications such as preeclampsia, HELLP syndrome, postpartum hemorrhage and abortions. Also, an important point to note here is that the background of pregnancy induced hypertension, preeclampsia and hemorrhage may act as a trigger to the development of the p-aHUS, especially in those with an underlying complement gene mutation.

These obstetric conditions do have overlapping and similar clinical and laboratory features to a certain extent. Hence, a high index of suspicion is essential for an early diagnosis of p-aHUS, that is, the triad of microangiopathic hemolysis, thrombocytopenia and renal involvement, in ante-partum as well as postpartum period, and also a prompt institution of treatment for better outcomes.

An interesting observation was the dismal results that were evident in studies from India and Pakistan, which could be attributed to the nonavailability of the terminal complement inhibitor, eculizumab in low- or middle-income countries/low-income countries, due to financial constraints.^[12,13]^ These studies have plasma exchange and plasma infusion as the primary treatment modality. The study by Ramachandran et al documented the highest maternal mortality rates of all the studies included.^[12]^ In terms of maternal morbidity, the ESKD risk was higher in the group not treated with eculizumab, again highlighting the complement-mediated nature of the disease.

Our data includes studies conducted after 2000, as majority of the data in the literature is beyond that period. Before 2000, very few case reports have been documented. Eculizumab is the monoclonal antibody targeting complement component C5, thereby blocking the uncontrolled activation of the alternative complement pathway, which is understood to be the pathogenesis of aHUS. FDA approved the use of eculizumab for aHUS in 2011,^[20,21]^ which has revolutionized the treatment and long-term outcomes of p-aHUS in developed countries. However, the availability of eculizumab in low- or middle-income countries/low-income countries has been difficult due to financial constraints, as seen in our data in studies from India and Pakistan. Some studies may be plagued by certain missing data and a lack of long-term follow-up.

Our study has many strengths. It is the most updated and diverse cohort of patients to develop p-aHUS. It is the largest compilation of patients with p-aHUS to have been treated with eculizumab and their long-term follow-up, compared to patients treated without the use of eculizumab. Our study has also included cases of p-aHUS with associated obstetric complications demonstrating the myriad clinical presentations of the disease, a vital aspect missing in international registry data on p-HUS.

Our data are limited by the design of studies, mainly case series, which provide abundant data but are biased by a lack of control data. Nonavailability of randomized controlled trials in the included cohorts is due to the nature of the disease and its rapid progression which warrants the best possible available treatment to every patient diagnosed at the earliest.

This data highlights the need for larger randomized controlled trials on p-aHUS. Also, more data on the use of newer complement blockade, Ravulizumab is needed. Widespread and equitable access to eculizumab in early therapy and long-term maintenance to all patients is the need of the hour, which we try to highlight by the outcomes of our study.

## Author contributions

**Conceptualization:** Priti Meena, Ruju Gala, Rashmi Ranjan Das, Vinant Bhargava, Yellampalli Saivani, Sandip Panda, Krishna Kumar Agrawaal.

**Data curation:** Priti Meena, Ruju Gala, Krishna Kumar Agrawaal.

**Formal analysis:** Priti Meena, Ruju Gala, Rashmi Ranjan Das, Vinant Bhargava, Yellampalli Saivani, Krishna Kumar Agrawaal.

**Investigation:** Priti Meena, Rashmi Ranjan Das, Yellampalli Saivani, Krishna Kumar Agrawaal.

**Methodology:** Priti Meena, Ruju Gala, Rashmi Ranjan Das, Vinant Bhargava, Yellampalli Saivani, Sandip Panda, Alok Mantri, Krishna Kumar Agrawaal.

**Project administration:** Priti Meena, Ruju Gala, Rashmi Ranjan Das, Alok Mantri, Krishna Kumar Agrawaal.

**Resources:** Priti Meena, Ruju Gala, Rashmi Ranjan Das, Yellampalli Saivani, Krishna Kumar Agrawaal.

**Software:** Priti Meena, Ruju Gala, Rashmi Ranjan Das, Vinant Bhargava, Yellampalli Saivani.

**Supervision:** Priti Meena, Vinant Bhargava, Yellampalli Saivani, Sandip Panda, Alok Mantri.

**Validation:** Priti Meena, Ruju Gala, Vinant Bhargava, Yellampalli Saivani, Alok Mantri.

**Visualization:** Priti Meena, Ruju Gala.

**Writing – original draft:** Priti Meena, Ruju Gala, Rashmi Ranjan Das, Vinant Bhargava, Yellampalli Saivani, Sandip Panda, Alok Mantri, Krishna Kumar Agrawaal.

**Writing – review & editing:** Priti Meena, Ruju Gala, Rashmi Ranjan Das, Vinant Bhargava, Yellampalli Saivani, Sandip Panda, Alok Mantri, Krishna Kumar Agrawaal.

## Supplementary Material



## References

[1] Goodship THJ Cook HT Fakhouri F; Conference Participants. Atypical hemolytic uremic syndrome and C3 glomerulopathy: conclusions from a “Kidney Disease: Improving Global Outcomes” (KDIGO) Controversies Conference. Kidney Int. 2017;91:539–51. 27989322 10.1016/j.kint.2016.10.005

[2] Fremeaux-Bacchi V Fakhouri F Garnier A. Genetics and outcome of atypical hemolytic uremic syndrome: a nationwide French series comparing children and adults. Clin J Am Soc Nephrol. 2013;8:554–62. 23307876 10.2215/CJN.04760512 PMC3613948

[3] Schifferli A Von Vigier RO Fontana M Spartà G Schmid H. Hemolytic-uremic syndrome in Switzerland: a nationwide surveillance 1997–2003. Eur J Pediatr. 2010;169:591–8. 19830454 10.1007/s00431-009-1079-9

[4] Bayer G Von Tokarski F Thoreau B. Etiology and outcomes of thrombotic microangiopathies. Clin J Am Soc Nephrol. 2019;14:557–66. 30862697 10.2215/CJN.11470918 PMC6450353

[5] Gupta M Govindappagari S Burwick RM. Pregnancy-associated atypical hemolytic uremic syndrome: a systematic review. Obstet Gynecol. 2020;135:46–58. 31809447 10.1097/AOG.0000000000003554 PMC6922068

[6] Dashe J. The long-term consequences of thrombotic microangiopathy (thrombotic thrombocytopenic purpura and hemolytic uremic syndrome) in pregnancy. Obstet Gynecol. 1998;91:662–8. 9572207 10.1016/s0029-7844(98)00031-3

[7] Rondeau E Cataland SR Al-Dakkak I Miller B Webb NJA Landau D. Eculizumab safety: five-year experience from the global atypical hemolytic uremic syndrome registry. Kidney Int Rep. 2019;4:1568–76. 31890998 10.1016/j.ekir.2019.07.016 PMC6933459

[8] Servais A Devillard N Frémeaux-Bacchi V. Atypical haemolytic uraemic syndrome and pregnancy: outcome with ongoing eculizumab. Nephrol Dial Transplant. 2016;31:2122–30. 27587606 10.1093/ndt/gfw314

[9] Gaggl M Aigner C Csuka D. Maternal and fetal outcomes of pregnancies in women with atypical hemolytic uremic syndrome. J Am Soc Nephrol. 2018;29:1020–9. 29282226 10.1681/ASN.2016090995 PMC5827586

[10] Bruel A Kavanagh D Noris M. Hemolytic uremic syndrome in pregnancy and postpartum. Clin J Am Soc Nephrol. 2017;12:1237–47. 28596415 10.2215/CJN.00280117 PMC5544502

[11] Huerta A Arjona E Portoles J. A retrospective study of pregnancy-associated atypical hemolytic uremic syndrome. Kidney Int. 2018;93:450–9. 28911789 10.1016/j.kint.2017.06.022

[12] Ramachandran R Nayak S Anakutti HP. Postpartum renal cortical necrosis is associated with atypical hemolytic uremic syndrome in developing countries. Kidney Int Rep. 2019;4:420–4. 30899869 10.1016/j.ekir.2018.11.012 PMC6409395

[13] Naqvi R. Hemolytic uremic syndrome associated with pregnancy: outcome from acute kidney injury. Pak J Med Sci. 2020;36:1153–7. 32968372 10.12669/pjms.36.6.2931 PMC7500999

[14] Timmermans SAMEG Werion A Spaanderman MEA. The natural course of pregnancies in women with primary atypical haemolytic uraemic syndrome and asymptomatic relatives. Br J Haematol. 2020;190:442–9. 32342491 10.1111/bjh.16626 PMC7496636

[15] Fakhouri F Scully M Ardissino G Al-Dakkak I Miller B Rondeau E. Pregnancy-triggered atypical hemolytic uremic syndrome (aHUS): a Global aHUS Registry analysis. J Nephrol. 2021;34:1581–90. 33826112 10.1007/s40620-021-01025-x PMC8494679

[16] Rondeau E Ardissino G Caby-Tosi MP. Pregnancy in women with atypical hemolytic uremic syndrome. Nephron. 2022;146:1–10. 10.1159/000518171 PMC8820436 34515154

[17] Korotchaeva Y Kozlovskaya N Shifman E Kudlay D Moiseev S. Eculizumab for pregnancy-related atypical hemolytic uremic syndrome. Nephrol Dial Transplant. 2024;39:1731–3. 38503569 10.1093/ndt/gfae068

[18] Fakhouri F Hourmant M Campistol JM. Terminal complement inhibitor eculizumab in adult patients with atypical hemolytic uremic syndrome: a single-arm, open-label trial. Am J Kidney Dis. 2016;68:84–93. 27012908 10.1053/j.ajkd.2015.12.034

[19] Legendre CM Licht C Muus P. Terminal complement inhibitor eculizumab in atypical hemolytic-uremic syndrome. N Engl J Med. 2013;368:2169–81. 23738544 10.1056/NEJMoa1208981

[20] Bortolotti M Barcellini W Fattizzo B. Molecular pharmacology in complement-mediated hemolytic disorders. Eur J Haematol. 2023;111:326–36 [published online ahead of print June 12, 2023]. 37308291 10.1111/ejh.14026

[21] European Medicines Agency Soliris (eculizumab). US Food and Drug Administration Soliris (eculizumab). https://www.ema.europa.eu/en/documents/product-information/soliris-eparproduct-information_en.pdf. Accessed June 30, 2024.

