# Supplementary material for: Kidney and pregnancy outcomes in pregnancy-associated atypical hemolytic uremic syndrome: A systematic review and meta-analysis
Source: Medicine (Baltimore). 2025 Jan 31;104(5):e41403. doi: 10.1097/MD.0000000000041403 (PMC11789862; doi:10.1097/MD.0000000000041403)
Supplement: Supplementary file 1 [file medi-104-e41403-s001.docx]

# Online supplementary data 1 Search terms for systematic review. Databases: Ovid MEDLINE

1. Kidney.mp
2. Renal.mp
3. Pregnancy /
4. Obstetrics /
5. 1 OR 2 AND 3 OR 4
6. Atypical Hemolytic Uremic Syndrome
7. 5 AND 7

# Databases: EMBASE:

“kidney” OR “renal” AND “Pregnancy" OR “Obstetrics” AND “Atypical Hemolytic Uremic Syndrome.

# Database: Cochrane Databases

Renal outcomes in pregnancy associated Atypical Hemolytic Uremic Syndrome

# Supplement Table S1: Newcastle-Ottawa quality assessment scale of included studies in meta-analysis

| Study | Selection | | | | Comparability | Outcome | | | Total score |
| --- | --- | --- | --- | --- | --- | --- | --- | --- | --- |
|  | Represent- ativeness | Selection of the non- exposed cohort | Ascertainment | Endpoint not present at start | Comparability | Assessment of outcome | Follow- up duration | Adequacy follow-up |  |
|  |  |  |  |  | (Confounding) |  |  |  |  |
| **Servais**  et al. | * | * | * | * | - | * | * | * | 5 |
| **Gaggl**  et al. | * |  | * | * |  | * | * | * | 6 |
| **Bruel et al** | * | * | * | * | * | * | * | * | 7 |
| **Huerta**  et al. | * | * | * | * | * | * | * | * | 7 |
| **Ramchandran** et al. | * |  | * | * |  | * | * | * | 6 |
| **Naqvi**  et al. | * |  | * | * |  | * | * | * | 6 |
| **Timmermans**  et al. | * | * | * | * | * | * | * | * | 7 |
| **Fakhori**  et al. | * | * | * | * | * | * | * | * | 7 |
| **Rondeau**  et al. | * |  | * |  |  | * |  |  | 3 |
| Korotchaeva et al. | * | * | * | * | * | * | * | * | 7 |

Notes: The Newcastle-Ottawa scale uses a star system (0 to 9) to evaluate included studies on 3 domains: selection, comparability, and outcomes. Star (*)= item presents. Maximum 1 star (*) for selection and outcome components and 2 stars (**) for comparability components. Higher scores represent higher study quality.

**Supplementary figure 1**
